# Surface Analysis of Native Spider Draglines by FE-SEM and XPS

**DOI:** 10.3389/fbioe.2020.00231

**Published:** 2020-03-20

**Authors:** Hiromitsu Sogawa, Kyohei Nakano, Ayaka Tateishi, Keisuke Tajima, Keiji Numata

**Affiliations:** ^1^Biomacromolecules Research Team, RIKEN Center for Sustainable Resource Science, Saitama, Japan; ^2^Emergent Functional Polymers Research Team, RIKEN Center for Emergent Matter Science, Saitama, Japan

**Keywords:** spider dragline, silkworm, silk fiber, FE-SEM, XPS, surface analysis

## Abstract

Although the physical and biological functions of the skin layer of spider dragline have been studied and partially clarified, the morphology and elemental contents of the skin layer of silk fibers have not been investigated in detail to date. Here, the surface of *Nephila clavata* spider dragline was evaluated by field emission scanning electron microscopy (FE-SEM) and X-ray photoelectron spectroscopy (XPS) to obtain clear surface morphological and molecular information. The FE-SEM images of the spider dragline indicate that the spider dragline forms a bundle of microfibrils. This hierarchical structure might induce faint fibrilar and network-like patterns on the surface of the dragline. XPS analysis revealed the presence of Na, P, and S, which are reasonably explained by considering the biological components of the major ampullate gland of spiders. The results obtained here are preliminary but will be important to consider the molecular transition of silk proteins to form excellent hierarchical structures during the spider dragline spinning process.

## Introduction

Silk fibers are well known as tough biomaterials, especially spider dragline silk, which possesses excellent toughness due to a combination of great tensile strength and ductility (Blackledge, 2012; Holland et al., 2018). The physical properties of dragline silk fibers depend not solely on protein constituents but also on the hierarchical microstructure (Sponner et al., 2007; Heim et al., 2010). Previously, it was hypothesized that the hierarchical structure of spider silk fiber is similar to that of silkworm silk fiber (Gosline et al., 1986; Vollrath, 1992). However, a highly organized skin-core structure of spider silk fiber was observed by atomic force microscopy (Li S.F. et al., 1994; Li S.F.Y. et al., 1994; Brown et al., 2012; Faugas et al., 2013) and light and electron microscopy (Thiel et al., 1994; Vollrath et al., 1996; Frische et al., 1998; Shao et al., 1999; Augsten et al., 2000), and this structure was different from the proposed structure of silkworm silk fiber (Gosline et al., 1986; Vollrath, 1992). Supercontracted spider silk fiber and the phenomenon of “supercontraction” were also used to investigate a structural hypothesis consisting of a fibril core surrounding an outer thin layer (Vollrath et al., 1996; Faugas et al., 2013). An experimental procedure to separate the different skin layers of dragline fibers has been developed (Sponner et al., 2007; Yazawa et al., 2019), and the results indicate that the order of the multilayer organization from exterior to interior is as follows: lipid coat, glycoprotein, skin, outer core, and inner core (Sponner et al., 2007). Additionally, Riekel et al. reported that the surface of the spider dragline consists of highly oriented protein chains forming an approximately 100 nm skin layer based on scanning transmission X-ray diffraction measurements (Rousseau et al., 2007; Riekel et al., 2017).

The skin layer is also important for the gas barrier properties of silk fibers. The mechanical and physical properties of spider dragline and silkworm silk fibers are known to depend on the humidity; namely, the amorphous and crystalline regions are influenced by water molecules (Fu et al., 2009; Yazawa et al., 2016; Malay et al., 2017). Spider dragline is a composite of multiple biopolymers that consists of a protein core surrounded by outer skin layers. Recently, we investigated the skin layer of *Nephila* spider dragline, especially the biological and physical functions of the skin layers (Yazawa et al., 2019). The mechanical/physical properties as well as the crystal structures do not depend significantly on the skin constituents, suggesting that the protein core region of spider dragline determines the structural and mechanical properties. Surprisingly, the skin layer does not influence supercontraction, i.e., the layer does not have water vapor barrier properties, but does protect the fiber from protease degradation activity. This result implies that the skin layer is more important as a biological barrier than a physical barrier.

Although the physical and biological functions of the skin layer of spider dragline have been studied and partially clarified, the morphological and elemental characterizations of the skin layer of silk fibers have not been investigated in detail to date. Here, the surface of the *Nephila clavata* spider dragline was evaluated by field emission scanning electron microscopy (FE-SEM) and X-ray photoelectron spectroscopy (XPS) to obtain clear surface morphological and molecular information.

## Experimental

### Collection and Preparation of Spider Dragline

Spider draglines (silk fibers) were collected from *Nephila clavata* females (body sizes: approximately 25 mm). Draglines were reeled at 21 mm/s and kept in lightproof boxes at a relative humidity from 30 to 50% to prevent UV damage and drying of the silk fibers.

### Preparation of Spider Silk Fibers Without a Skin Layer

The removal of the skin layer was conducted according to a previously reported method (Sponner et al., 2007; Yazawa et al., 2019). Briefly, *N. clavata* spider dragline silk fibers were immersed twice in diethyl ether for 10 min. To remove the diethyl ether, the fibers were dried overnight in a vacuum oven at 40°C.

### Field Emission Scanning Electron Microscopy

The surface morphology of the spider dragline was observed and characterized by field emission scanning electron microscopy (FE-SEM, GeminiSEM, Carl Zeiss, Oberkochen, Germany). The silk fiber samples were mounted on an aluminum stub with conductive tape without sputter-coating prior to FE-SEM visualization. For the observation, the acceleration voltage and working distance were set at 0.5–1.0 kV and 2.0 mm, respectively.

### Enzymatic Treatment

For the enzymatic treatment, we used the native draglines without the diethyl ether wash. The native draglines were immersed in 0.1 M phosphate buffer (pH = 7.4) containing 0.5 mg/mL of proteinase K. Less than 1 mg of spider dragline was immersed in 1 mL of the enzyme solution at 37°C for 24 h. The enzymatic degradation conditions were determined based on previous reports on enzymatic degradation (Li et al., 2003; Numata et al., 2005, 2008, 2010; Numata and Kaplan, 2010). After the enzymatic treatment, the sample was washed with 0.1 M phosphate buffer and Milli-Q water twice and dried for 1 day under vacuum conditions.

### XPS Measurements

X-ray photoelectron spectroscopy (XPS) was performed with a photoelectron spectroscopy system (PHI 5000 Versa Probe II, ULVAC-PHI). Monochromated Al Kα (1486.6 eV) radiation with an operating power of 50 W (15 kV voltage) was used in all the XPS measurements. The analyzed area was 200 μm. The XPS survey spectra (Figure 3B) were measured with a pass energy of 117.4 and 0.125 eV energy step. For the measurement of each atomic element, pass energy of 23.5 and 0.025 eV energy steps were used. The recorded signals were accumulated eight times for C1s, O1s, and twelve times for other elements. A take-off angle was 45° to the sample substrate. During the measurement, the samples were neutralized using both a low-energy ion beam and a low-energy electron beam.

For data analysis, we used PHI MultiPak software (ULVAC-PHI). The signal background of each component was subtracted using the Shirley method. The atomic concentration was calculated by considering the relative sensitivity factor for each element corrected with the sensitivity factor of the system. The fitting protocol used the Gaussian-Lorentzian function, but the best fit results were obtained with Gaussian 100%.

## Results and Discussion

### FE-SEM Observations of Silk Fibers

**FIGURE 1:**
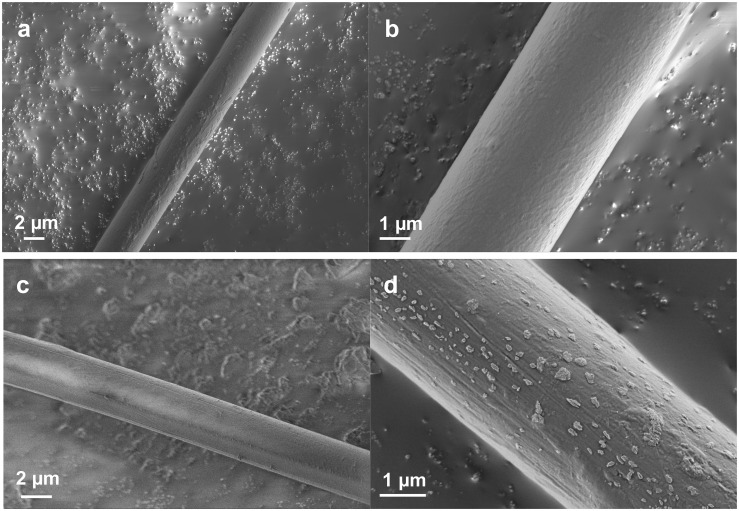
FE-SEM images showing the surface of silk fibers at different magnifications. **(a,b)** Native *Nephila* spider dragline. **(c,d)** *Nephila* spider dragline after ether extraction.

FE-SEM observations have several advantages over general SEM imaging. In this study, direct imaging of a single dragline without a sputter-coating process was conducted to observe the surface morphology at high resolution. Figure 1 shows the typical FE-SEM images of the native *Nephila* dragline and the dragline without a skin layer. In previous literature with SEM imaging (Lin et al., 2017; Yazawa et al., 2019), the surface of spider draglines has been reported to be smooth and clean. However, we found with FE-SEM that the surface of the *Nephila* spider dragline has fibril and network-like patterns (Figure 1b). Once the silk layer was washed out with ether, the patterns disappeared, but debris from the skin layer was observed at the fiber surface (Figures 1c,d). The white spots indicate the residues of the lipid layer, which is the outermost layer of dragline silk. As a comparison, the surface morphologies of silkworm silk, which were previously reported and summarized, were referenced (Malay et al., 2016). In many cases, inorganic compounds have been observed on the surface of silkworm cocoon silk fibers (Numata et al., 2015). These previous reports on the surface morphologies of silkworm silks differ from the patterns found on the surface of the spider dragline. In this study, the silk fibers were not pretreated for FE-SEM imaging, and hence, the surface morphologies should not be induced by the sample treatment. Therefore, the natural spider dragline seems to show characteristic fibril and network-like patterns. The patterns might be related to the spinning process of spiders, especially the final part around the spinneret.

### Enzymatic Treatment

**FIGURE 2:**
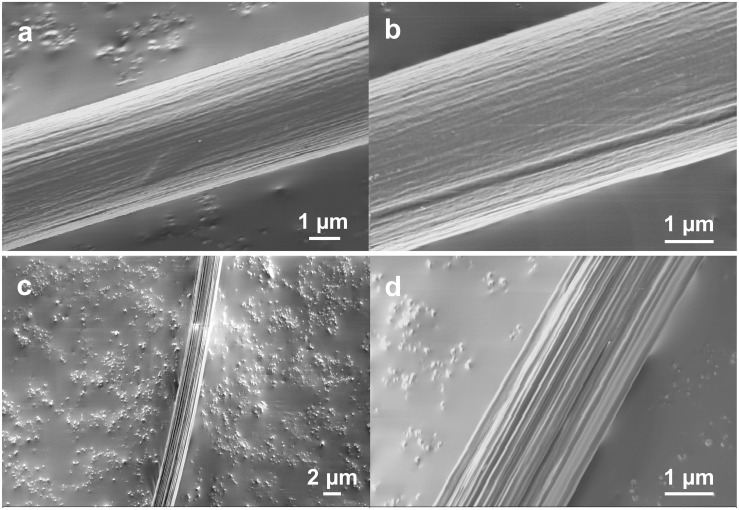
FE-SEM images showing the surface of *Nephila* spider dragline after proteinase-K treatment. **(b,d)** Are enlarged images of **(a,c)**, respectively.

To further examine the surface morphologies, especially the fibril and network-like patterns, we studied the morphological changes of the native *Nephila* spider dragline after enzymatic treatment with proteinase K. We treated the spider dragline with proteinase K (without any diethyl ether treatment), an efficient protease, and subsequently observed the dragline by FE-SEM (Figure 2). To avoid misunderstanding of the biodegradability of the spider dragline, we need to clear the biodegradability of spider dragline, according to the previous study (Yazawa et al., 2019). Yazawa et al. (2019) reported that the skin layer of the dragline can protect the dragline from the enzymatic attacks. Proteinase K, which was found in extracts of the fungus *Engyodontium album*, is a serine protease with a broad substrate specificity and high activity against aliphatic and aromatic amino acids. The biodegradation of the spider dragline by proteinase K is not expected in the natural environments, because *Nephila* spider does not prefer to construct web and territories in the environments contaminated with fungi.

The enzymatic degradation behavior of the dragline was not consistent, as Figures 2a,c show different degradation degrees of the draglines. Figures 2a,b clearly shows that the dragline consists of a bundle of microfibrils. Furthermore, Figures 2c,d indicates that the bundle structures of microfibrils are maintained even inside of the dragline. Thus, both FE-SEM images indicate that the spider dragline forms a structure similar to a bundle of microfibrils. The enzymatic treatment degraded the skin layer and silk fibroins of the native draglines, resulting that these microfibrils were exposed and observed by FE-SEM. This hierarchical structure might induce faint fibril patterns at the surface of the dragline, however, we need to further study the effect of the natural spinning process with spider spinnerets.

### XPS Measurements

**FIGURE 3:**
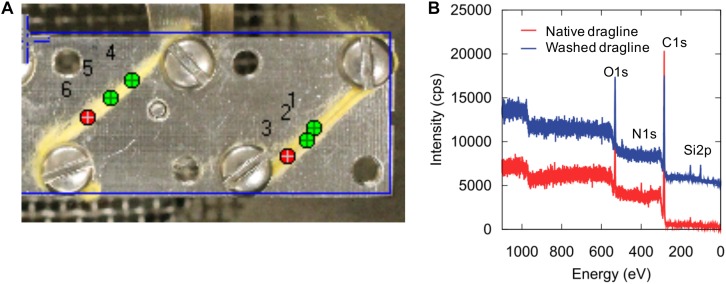
XPS sample setting **(A)** and wide-scan spectra **(B)** of native spider dragline (red) and spider dragline after ether wash (blue).

**FIGURE 4:**
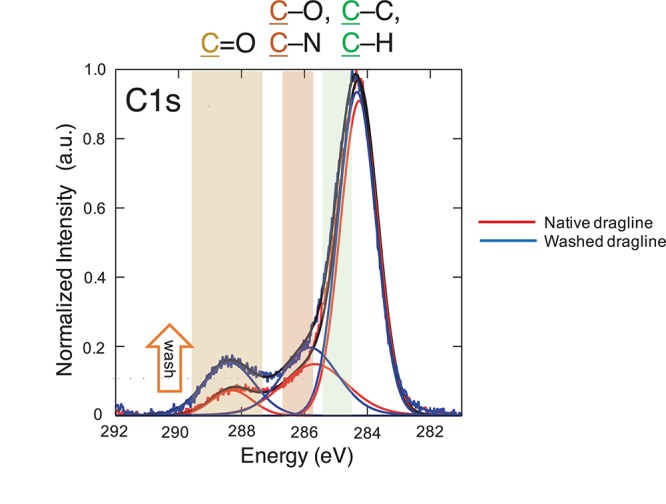
XPS spectra of the native spider dragline (red) and spider dragline after washing with ether (blue). The peak fitting results are also shown in the same color. The details on the peak fitting is described in Experimental section.

**FIGURE 5:**
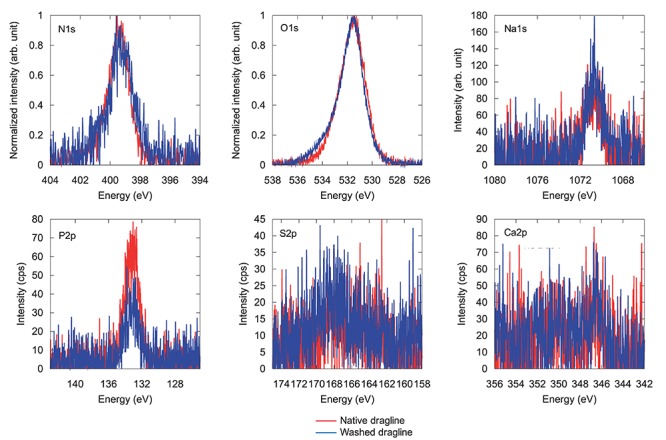
XPS spectra for different elements in native spider dragline (red) and spider dragline after an ether wash (blue).

XPS is a powerful method for characterizing the surface chemical composition and chemical states of each element. An XPS study of recombinant silk protein during a thermal degradation process was reported previously (Dao et al., 2017), but natural spider draglines have not been investigated to date. Figure 3 shows the setting of the fiber samples and the wide-range XPS spectra of the native dragline and the dragline washed with diethyl ether for skin layer removal. The peaks originating from silicon were unexpected contamination from the washing experiment. We could not detect potassium in the wide-range XPS spectra, even though the presence of potassium is expected according to the previous ionic elemental analyses of the major ampullate glands (Knight and Vollrath, 2001; Andersson et al., 2014; Oktaviani et al., 2019). Perhaps, potassium might not be localized at the surface of the dragline. On the other hand, O 1s, C 1s, and N 1s were obviously detected as shown in Figures 4, 5.

The spectra concerning C 1s were further assigned as shown in Figure 4, namely, C = O, C-O/C-N, and C-C/C-H. The XPS spectra were deconvoluted with the Gaussian–Lorentzian mixed function to resolve multiple chemical states, as listed in Table 1. The standard deviations of the contents include all the errors from the variation of the atomic concentration of the samples, positional accuracy of the fitting, and also from the equipment itself. We think the primary origin of the deviation might be the variation of the samples themselves because our samples are natural ones. The increase in C = O content suggests the appearance of peptide bonds at the surface after the washing process. The removal of the lipid layer via the diethyl ether treatment was successfully detected by XPS. Thus, the XPS measurements with spider draglines indicated sufficient quantification to discuss the surface chemical compositions.

**TABLE 1:** XPS characterization of different elements and components of native spider dragline and spider dragline after an ether wash.

**% (Standard deviation)**	**C 1s C-H**	**C 1s C = O**	**C 1s C-O**	**N 1s**	**O 1s**	**Na 1s**	**P 2p**	**S 2p**	**Ca 2p**
Native dragline	60.1 (6.3)	6.8 (2.3)	16.0 (2.5)	2.8 (1.4)	8.8 (2.7)	1.2 (0.7)	0.68 (0.06)	0.53 (0.16)	0.75 (0.45)
Washed dragline	50.1 (2.6)	8.2 (2.1)	16.3 (3.2)	2.4 (0.1)	13.5 (3.7)	1.4 (0.5)	0.48 (0.25)	0.74 (0.08)	1.0 (0.53)

*Numbers in parentheses are standard deviations in results obtained three different measurement positions.*

Figure 5 shows each spectrum corresponding to N 1s, O 1s, Na 1s, P 2p, S 2p, and Ca 2p. Only phosphate, P 2p, decreased in intensity after the skin layer removal. Considering that phosphate ions exist in the overall ampullate gland of spiders (Andersson et al., 2014; Oktaviani et al., 2019), the skin layer may contain more phosphate ions than the protein core inside the dragline. The peak corresponding to calcium, Ca 2p, could be background noise because of the abundant presence of calcium ions in the natural environment. The other elements, N 1s, O 1s, Na 1s, and S 2p, did not show any significant change before and after the skin removal treatment. Sodium should be related to the ionic conditions of the spider ampullate gland, but we could not find any related difference between the skin layer and core regions. The S 2p peak, which can be related to sulfate ions and the cysteines of spider silk proteins, was detected even though its intensity and signal-to-noise ratio were very low. The sulfate ion is kosmotropic and can be present at the end of the spider major ampullate gland to form the dragline; hence, it is reasonable to detect the S 2p peak by XPS. However, we gave up discussing more details of the S 2p peak, due to the relatively low intensity. The Ca 2p peak can be related to Ca ions, which are predominantly present under the natural environments. Similar to the S 2p peak, we avoid to discuss the more details of the Ca 2p based on the current signal-to-noise ratios.

## Conclusion

We evaluated the surface of *Nephila clavata* spider dragline by FE-SEM and XPS to obtain clear surface morphological and chemical information. This is the first surface analysis of natural spider draglines by both powerful techniques. The FE-SEM images of the spider dragline indicate that the spider dragline forms a bundle of microfibrils. This hierarchical structure might induce faint fibril and network-like patterns at the surface of the dragline, however, we need to further study the origin of the patterns. Perhaps, we need to consider the effect of the natural spinning process with spider spinnerets to explain the surface patterns found in this study. XPS analysis revealed the presence of Na, P, and S, which are reasonably explained by considering the biological components of the major ampullate gland of spiders. The information obtained here is preliminary but will be important and essential to consider the molecular transition of silk proteins to form excellent hierarchical structures during the spider dragline spinning process.

## Data Availability Statement

The raw data supporting the conclusions of this article will be made available by the authors, without undue reservation, to any qualified researcher.

## Author Contributions

HS prepared the samples. AT conducted the FE-SEM measurements. KNa performed the XPS experiments. KT and KNu conceptualized and oversaw the project. All the authors co-wrote the manuscript.

## Conflict of Interest

The authors declare that the research was conducted in the absence of any commercial or financial relationships that could be construed as a potential conflict of interest.
